# Metastasis to Paranasal Sinuses from Carcinoma of Prostate: Report of a Case and Review of the Literature

**DOI:** 10.1155/2018/5428975

**Published:** 2018-03-25

**Authors:** Elio Bittar Barbosa, Evaldo César Macau Furtado Ferreira, Fernanda Viviane Mariano, Albina Messias de Almeida Milani Altemani, Emerson Taro Inoue Sakuma, Eulalia Sakano

**Affiliations:** ^1^Department of Ophthalmology and Otorhinolaryngology, Faculty of Medical Sciences, University of Campinas, Campinas, SP, Brazil; ^2^Department of Pathology, Faculty of Medical Sciences, University of Campinas, Campinas, SP, Brazil; ^3^Department of Radiology, Faculty of Medical Sciences, University of Campinas, Campinas, SP, Brazil

## Abstract

Metastasis from distant primary tumors is extremely rare in the paranasal sinuses with few hundred cases in the literature. Metastatic carcinoma of the prostate is even rarer, despite being one of the most common tumors, with only 24 cases published. In this article, we report a case of a 58-year-old male presenting with epistaxis and nasal obstruction as initial symptoms of a metastatic prostate carcinoma in the ethmoid cells and maxillary sinus.

## 1. Introduction

Paranasal sinus cancer represents a small portion of the head and neck cancer, approximately 5% of all head and neck tumors [1]. These tumors may arise from multiple tissues present in the nose and paranasal sinuses [2] or more rarely be a metastasis from a distant primary cancer.

Prostate adenocarcinoma is the most prevalent malignant cancer in the male population. In Europe, the incidence rate is 214 cases per 1000 men [3]. Metastases from prostate tumors are a major determinant of survival rates, and prostate-specific antigen tests decrease the incidence of metastatic disease at diagnosis [4].

In this article, we presented the rare case of a patient with a distant metastasis in the right ethmoid cells from a prostate adenocarcinoma.

## 2. Case Report

A 58-year-old male patient was referred to the Otolaryngology Emergency Service because of important unilateral epistaxis, the third episode in the previous month. He also presented with ipsilateral nasal obstruction and diplopia with a three-month evolution. In his medical report, there was a record of penile squamous cell carcinoma treated with surgery and radiotherapy in 2014 and stage IV prostate adenocarcinoma (iliac, lumbar, and encephalic metastasis) refractory to hormone therapy and chemotherapy, submitted to palliative radiotherapy one month earlier.

During endoscopy, a polypoid, papilloma-like mass originated from the middle meatus was visible, occupying the entire right nasal fossa. No other abnormality was seen in the left nasal fossa. The epistaxis was controlled; the patient was stabilized and referred to biopsy.

**Figure 1:**
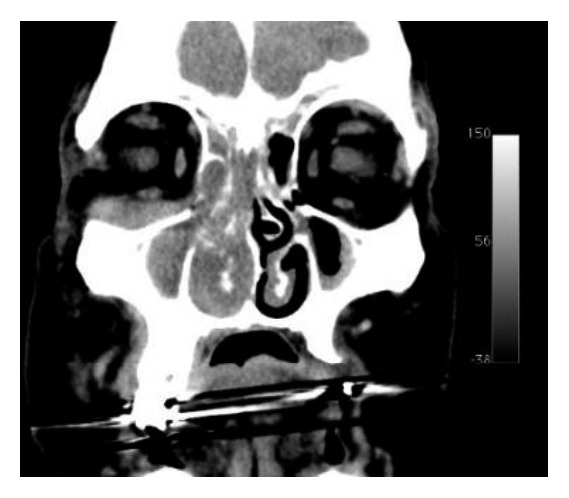
CT demonstrates lesion occupying ethmoid cells, nasal cavity, and maxillary sinus and extending through the lamina papyracea.

**Figure 2:**
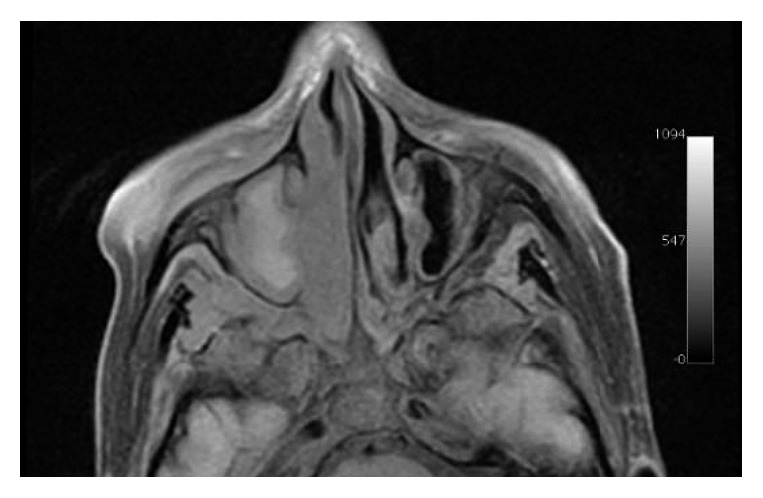
T1-weighted MRI with fat suppression demonstrates the lesion extending to the pterigomaxillary fissure.

The computed tomography (CT) and magnetic resonance imaging (MRI) showed soft tissue density lesion in the right nasal fossa, ethmoid cells, and maxillary sinus with extension into the inferior portion of the orbit through the lamina papyracea and posteriorly to the pterygomaxillary fissure (Figures 1 and 2).

**Figure 3:**
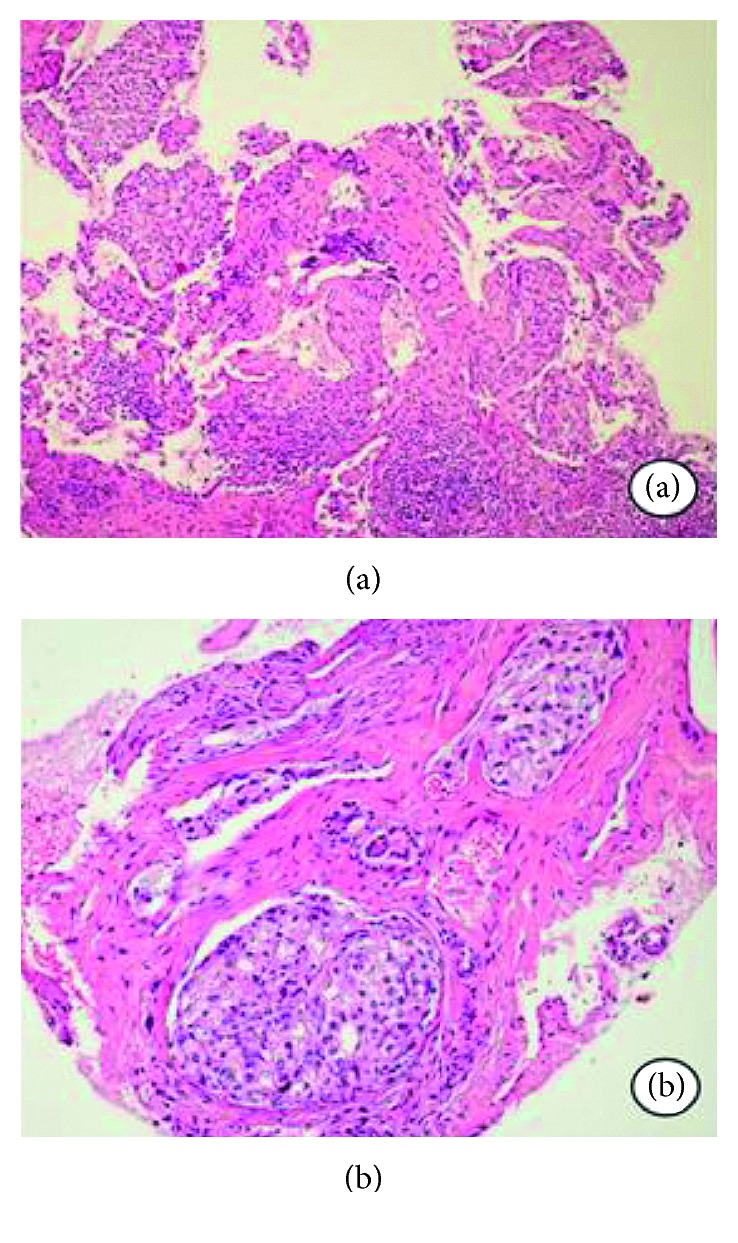
(a) Neoplasia infiltrating surrounding tissue with irregular margins (H&E stain). (b) Atypical epithelial islands with ductal characteristics resembling adenocarcinoma (H&E stain).

**Figure 4:**
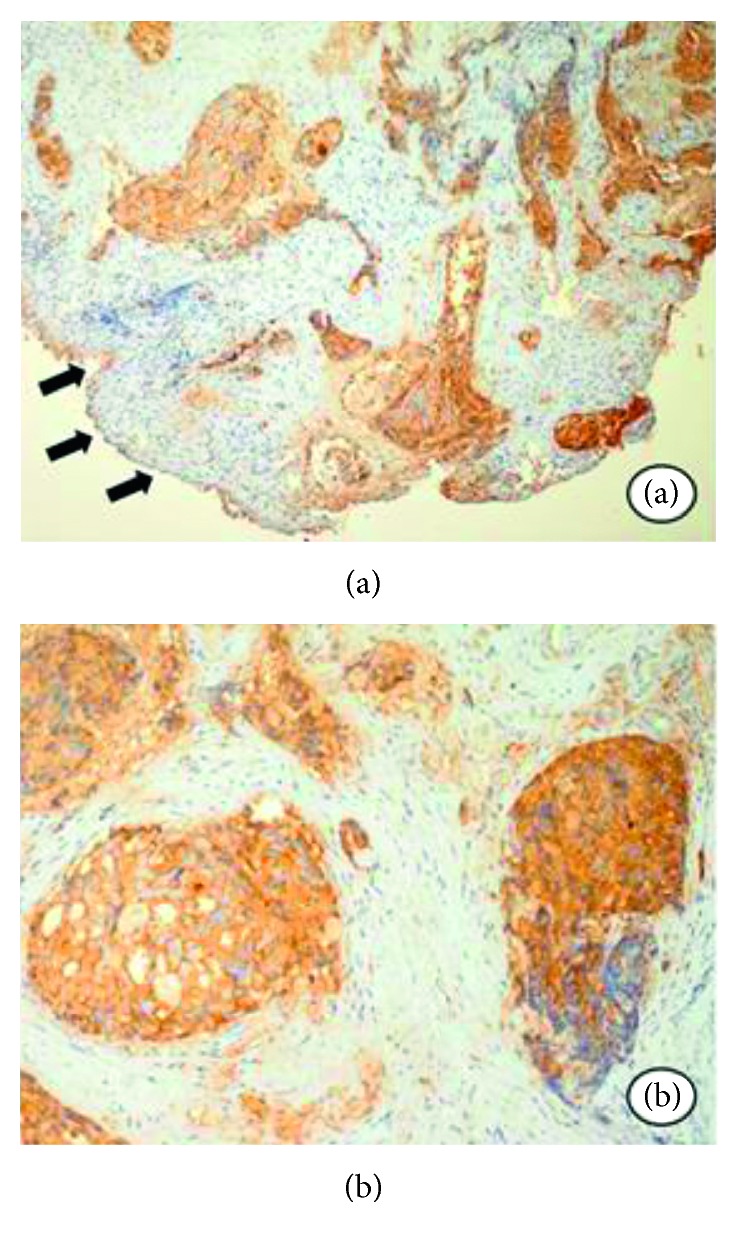
(a) Positive reaction to the PSA antigen in the epithelial islands, and there is no reaction in the superficial epithelium. (b) Strong expression of the PSA antigen in the ductal cells.

The biopsy resulted in adenocarcinoma (Figures 3(a) and 3(b)). Since the patient had a medical report of prostate adenocarcinoma, the sample was submitted to an immunohistochemical panel. The prostate-specific antigen (PSA) was highly positive, and the diagnosis was confirmed as metastatic prostate adenocarcinoma (Figures 4(a) and 4(b)).

Because of the clinical status of the patient, a few symptoms presented, and the fact that he was already submitted to radiotherapy at the same field, it was opted for a clinical follow-up. After two months of the diagnosis, the patient deceased.

## 3. Discussion

Paranasal sinuses are a complex anatomic area, surrounding important structures such as the orbit and skull base. The most incident tumors are the squamous cell carcinoma, followed by adenocarcinoma and adenoid cystic carcinoma [5].

In a review, Prescher and Brors reported 169 cases of metastatic tumor to the paranasal sinuses [6]. Most cases originated from the kidney, followed by the lung, breast, thyroid, and prostate. Prescher and Brors also reported that the maxillary sinus was the most affected, followed by the sphenoid, ethmoid, and frontal sinuses [6]. These data are similar to those published by Bernstein [7]. In 77% of the cases, just one paranasal sinus was affected [6].

The nonspecific symptoms are similar to those of primary tumors. The nasal symptoms are usually nasal mass, nasal obstruction, facial deformity, and epistaxis. Orbital symptoms may also occur, such as proptosis, ptosis, decreased vision, and diplopia. Occasionally, these symptoms may be the first presentation of an occult primary tumor [8, 9].

The most frequent sites involved in prostate metastasis are the bone (90%), lung (46%), and liver (25%) [10]. The head and neck are rare locations for metastasis, and it occurs more frequently in the brain, dura, and lymph nodes [11]. The treatment may be hormone therapy, chemotherapy, radioisotopes, and radiotherapy [12].

Metastasis may reach the paranasal sinuses by hematogenous, lymphogenous, or vertebral venous plexus pathways. First postulated by Batson [13], this low-pressure valveless system is a connection between deep pelvic veins, intercostal veins, vena cava, and the azygos system. A rise in the abdominal pressure might redirect the blood flux from the vena cava system to the vertebral venous plexus. This flux alteration can allow the tumor to reach the paranasal sinuses [14].

This is the twenty-fifth reported case of metastatic prostate adenocarcinoma in the paranasal sinuses. The majority of cases involved patients with known prostate cancer, with a mean age of 63.15 years, and the most affected sinus was the sphenoid (44.5%). Radiotherapy was the preferred treatment option (40.7%). In Table 1, we describe the cases reported in full text, available online so far.

Imaging is not able to differentiate a local tumor from a metastasis; however, it is essential to determine location and extension and for surgical planning. CT may show enhancement, bone erosion, remodeling, and invasion. Magnetic resonance imaging (MRI) has an important role to help, defining leptomeningeal and orbital invasion [13]. Positron emission tomography (PET-CT) might be useful in the primary occult tumor.

Histopathology has an essential role in the diagnosis. When metastasizing to the paranasal sinuses, normally prostate carcinoma is not well differentiated. Immunohistochemical panel is also important, with positivity of prostate-specific antigen, prostate acid phosphatase, EpCam, NKX3.1, and prostein [36].

Usually, the diagnosis of a metastasis to the paranasal sinuses means a poor prognosis. An important factor that can be crucial is whether the metastasis is isolated or part of a widespread disease.

Normally, the treatment involving the metastasis to the paranasal sinuses is palliative, with the exception of an isolated metastasis, for which the radical surgery may be a viable option. The patient's quality of life should be a priority. The main goal is pain relief and bleeding prevention. Management has not changed greatly over the years, and therapy options include radiotherapy, chemotherapy, immunotherapy, and, more recently, endoscopic surgery [37].

Endoscopic surgery may provide symptom relief faster, with lower systemic and local side effects [37]. Tabaee et al. suggested three criteria to help in the decision of the surgery: reasonable expectation of improvement, possible prolongation of life, and survivability after anesthesia [38].

## 4. Conclusion

Despite being a rare entity, metastatic prostatic tumor in the paranasal sinuses should always be part of the differential diagnosis in patients with known prostatic tumor and recently developed nasal or orbital symptoms.

## Figures and Tables

**Table 1:** Prostate metastasis to the paranasal sinuses.

Author	Age	Sinus	Symptoms	Treatment	Survival
Barrs et al. [15]	61	Sphenoid	Diplopia	Unknown	Died 2 years after presentation
Barrs et al. [15]	57	Sphenoid	Diplopia, decreased visual acuity, ptosis, and numbness of the left face	Unknown	Died 2 years after presentation
McClatchey et al. [16]	54	Sphenoid	Frontal headache and blurring of the right eye	Radiotherapy	Alive 1 year after presentation
Leduc et al. [17]	75	Sphenoid	Diplopia and ptosis of the right eye	Pulpectomy	Alive 19 months after presentation
Matsumoto et al. [18]	79	Sphenoid	Headache and diplopia	Orchiectomy	Unknown
Har-el et al. [19]	77	Maxillary	Exophthalmos of the right eye	Orchiectomy and hormonal block	Unknown
Mickel and Zimmerman [20]	67	Sphenoid	Diplopia and numbness on the right side of the nose	Radiotherapy	Died 2 and a half months after presentation
Saleh et al. [21]	71	Sphenoid	Bilateral exophthalmos and hemoptysis	None	Died 1 month after biopsy
Fortson et al. [22]	50	Ethmoid	Nasal obstruction, diplopia, proptosis, and blurred vision	Chemotherapy and radiotherapy	Died 7 months after presentation
Telera et al. [23]	61	Sphenoid	Ptosis and diplopia of the right eye	Radiotherapy	Died 13 months after presentation
Oliver et al. [8]	72	Maxillary, frontal, and ethmoid	Frontal headache and retro-orbital pain	Hormonal block	Alive three months after presentation
Hunt et al. [11]	76	Sphenoid	Unknown	Radiotherapy and hormonal block	Alive 14 months after presentation
Lavasani et al. [24]	67	Sphenoid	Decreased visual acuity	Radiotherapy	Alive 6 months after presentation
Başeskioglu et al. [25]	69	Maxillary	Sinus fullness	Radiotherapy	Died 32 months after diagnosis
Ibarguren et al. [26]	64	Maxillary and frontal	Ptosis, proptosis, and facial numbness	Hormonal block and chemotherapy	Alive 8 months after presentation
El Khatib et al. [27]	57	Maxillary	Facial swelling	Pulpectomy and hormonal block	Died 9 months after presentation
Viswanatha [28]	68	Ethmoid and frontal	Facial swelling and epistaxis	Radiotherapy	Lost to follow-up after 3 months of presentation
Tunio et al. [29]	65	Ethmoid	Nasal obstruction, diplopia, and proptosis	Radiotherapy and hormonal block	Alive until article publication
Azarpira et al. [30]	74	Maxillary	Nasal obstruction	Chemotherapy and radiotherapy	Died 11 months after presentation
Petersson et al. [31]	55	Sphenoid	Headache, diplopia, and blurred vision	Hormonal block	Unknown
Puche-Sanz et al. [32]	56	Sphenoid	Decreased visual acuity and facial numbness	Radiotherapy and hormonal block	Alive 5 years after presentation
Akdemir et al. [33]	73	Frontal and ethmoid	Headache and exophthalmos	Hormonal block	Unknown
Evarts et al. [34]	59	Maxillary and ethmoid	Cheek numbness, headache, decreased visual acuity, nasal obstruction, and drainage	Chemotherapy	Died 2 months after biopsy
Lechien et al. [35]	67	Frontal	Diplopia, facial pain, and headache	Hormonal block	Died a few months after presentation
Present case	58	Ethmoid and maxillary	Epistaxis, nasal obstruction, and diplopia	None	Died 2 months after diagnosis
